# Effects of a Short Educational Program for the Prevention of Foot Ulcers in High-Risk Patients: A Randomized Controlled Trial

**DOI:** 10.1155/2015/615680

**Published:** 2015-09-10

**Authors:** Matteo Monami, Stefania Zannoni, Marianna Gaias, Besmir Nreu, Niccolò Marchionni, Edoardo Mannucci

**Affiliations:** ^1^Division of Geriatric Cardiology and Medicine, Careggi Teaching Hospital, 50141 Florence, Italy; ^2^Division of Diabetes Agency, Careggi Teaching Hospital, 50141 Florence, Italy

## Abstract

*Background*. Patient education is capable of reducing the risk for diabetic foot ulcers. However, specific education on foot ulcer prevention was either included in broader programs addressing different parts of diabetes care or provided with time- and resource-consuming curricula. The aim of the study is to assess the feasibility and efficacy of a brief educational program for the prevention of diabetic foot ulcers in high-risk patients.* Methods*. The study was performed on type 2 diabetic patients, randomized in a 1 : 1 ratio either to intervention or to control group. The principal endpoint was the incidence of foot ulcers. The intervention was a two-hour program provided to groups of 5–7 patients, including a 30-minute face-to-face lesson on risk factors for foot ulcers, and a 90-minute interactive session with practical exercises on behaviors for reducing risk.* Results*. The study was prematurely terminated due to a highly significant difference in outcome between the two treatment groups. The final sample was therefore composed of 121 patients. Six patients, all in the control group, developed ulcers during the 6-month follow-up (10% versus 0%, *p* = 0.012).* Conclusions*. A brief, 2-hour, focused educational program is effective in preventing diabetic foot ulcers in high-risk patients.

## 1. Introduction

Several studies, including some randomized controlled trials [1, 2], have shown that patient education is capable of reducing the risk for diabetic foot ulcers [3]. However, specific education on foot ulcer prevention is often included in broader programs addressing different parts of diabetes care [1–6], or provided with time- and resource-consuming curricula [2]. Some trials have explored the efficacy of dedicated educational interventions for prevention of foot ulceration, usually with individual sessions [7–10]; some of those trials [7, 8] had a short-term follow-up, providing no data on the effect of the intervention on the incidence of new ulcers. One trial had a long-term (7-year) follow-up, but the patients enrolled had a low risk of foot ulcers, so that the number of events observed was not sufficient to draw clear conclusions [10].

Group educational programs could theoretically be more cost-effective than individual patient education. In a randomized trial, a 1-hour group educational program reduced the incidence of amputation and new ulcerations in diabetic patients with foot infection, ulceration, or prior amputation referred for podiatry or vascular surgery [11]. Though interesting, the results of that latter trial cannot be easily extended to patients with a lower risk profile.

Considering that available resources are limited, brief and inexpensive educational programs have a greater chance of being applied in routine clinical practice. Although longer and more intensive educational programs were reported to be more effective than brief interventions for foot ulcer prevention [2], other studies failed to detect significant differences between these two approaches [4, 5]. A properly designed brief program could produce some beneficial effect with a limited use of resources. In addition, the selection of patients at higher risk could be crucial for cost-effectiveness of patient education.

The aim of the present study is to assess the feasibility and efficacy of a brief educational program for the prevention of diabetic foot ulcers in high-risk patients referring to a diabetes outpatient clinic.

## 2. Patients and Methods

This study, designed as a randomized, open-label, single-center clinical trial, with a 6-month follow-up, was approved by the local Ethical Committee. The study was performed on outpatients aged ≥18 years, affected by type 2 diabetes, who fulfilled at least one of the following three criteria (for definition of high risk of foot ulcers): diagnosis of neuropathy, previous diabetic foot ulcer, or foot abnormalities at risk for ulcer in the opinion of the investigator. Patients with peripheral vascular disease requiring immediate revascularization, as well as those with cognitive impairment, were excluded. All patients had previously received standard multidisciplinary education for diabetes (with a structured group program at diagnosis or first contact, and follow-up meetings every two years), but no educational intervention specifically focused on foot care. After providing written informed consent, patients were randomized in a 1 : 1 ratio either to intervention or to control group. The randomization procedure was based on a computer-generated list held by an independent randomization center (Diabetes Agency) that was contacted by telephone each time a person was randomized. The principal endpoint was the incidence of foot ulcers.

The intervention was a two-hour program provided to groups of 5–7 patients (mean: *n* = 6), including a 30-minute face-to-face lesson on risk factors for foot ulcers, and a 90-minute interactive session with practical exercises on behaviors for reducing risk. The intervention involved a physician (for 15 minutes) and a nurse (for the remaining 105 minutes). A detailed description of the curriculum can be found in the appendix. Patients randomized to control group were provided with a brief leaflet with some recommendations for ulcer prevention, as suggested by local guidelines [6].

At randomization, the PIN (Patient Interpretation of Neuropathy) questionnaire was administered to the patients, exploring patients' knowledge about signs and symptoms of neuropathy and risk factors for foot ulcers onset [11]. In patients randomized to intervention, the questionnaire was administered again at the end of the educational session.

Follow-up visits were planned at 3 and 6 months from randomization, for foot examination. Patients who did not show up at control visits were actively contacted through telephone calls. In patients who developed ulcers, the number of visits at the foot clinic (performed either by physician or by nurse) was recovered from administrative databases of hospital activity; physicians' and nurses' visits are scheduled every 30 and 20 minutes, respectively.

The power calculation, based on the incidence of ulcers observed in previous studies [7], suggested the enrolment of 100 patients per group to detect a 20% between-group difference (power 80%, *p* < 0.05, and drop-out 2%).

For statistical analysis, continuous variables (expressed as mean ± SD or as median [quartiles]) were compared between groups with unpaired Student's *t*-tests or Mann-Whitney *U* tests, whenever appropriate. Chi-square test was used for between-group comparisons of categorical variables. Relative risk of incident foot ulcers (with 95% confidence interval, 95% CI) was calculated using Kaplan-Meier method.

## 3. Results

The study was prematurely terminated due to a highly significant difference in outcome between the two treatment groups. The final sample was therefore composed of 121 patients. One patient (in the intervention group) was lost at follow-up and was therefore excluded from the analysis. The baseline characteristics of the final sample are summarized in Table 1, and they did not differ between the two groups. Patients allocated to the intervention group showed a trend toward reduction of HbA1c and BMI at 6 months, which did not reach statistical significance, whereas blood pressure levels did not show any change (Table 2).

**Figure 1:**
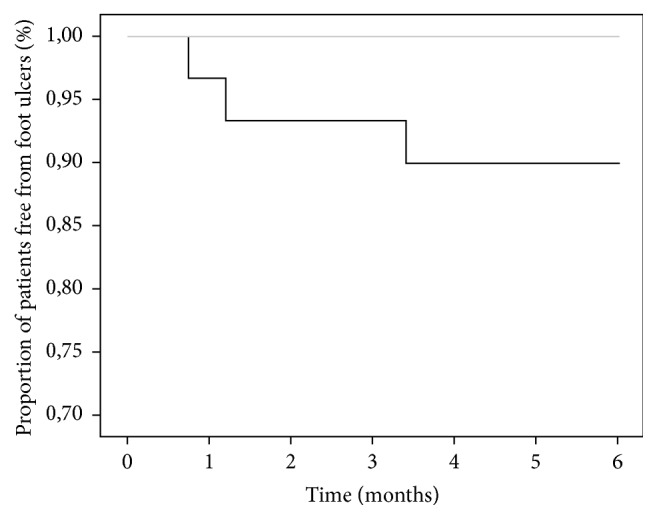
Kaplan-Meier survival curves (patients free of event) for incidence of foot ulcers in intervention (grey line) and control (black line) groups. *p* = 0.012.

No amputation was reported in the sample enrolled. Two patients died during follow-up (one in the standard care and one in the interventional group); none of them had developed ulcers, and they were included in the analysis until death. Six patients, all in the control group, developed ulcers during the 6-month follow-up (10% versus 0%, *p* = 0.012; Figure 1). Questionnaire scores improved significantly after intervention (20 [16; 22] versus 23 [21; 24], *p* < 0.001). No statistical difference in questionnaire score at baseline was detected between the two groups.

Time spent for intervention was 150 and 1050 minutes (2.5 and 17.5 minutes per patient) for physician and nurse, respectively, whereas time spent for ulcer care in the control group was 390 and 1200 minutes (6.5 and 20 minutes for each patient randomized to control).

## 4. Discussion

A brief, 2-hour, focused group educational program is effective in preventing diabetic foot ulcers in high-risk patients, as previously reported in another study [2]. With respect to the trial by Malone et al. [11], the present study was performed on patients with a lower overall risk profile, referring to a diabetes outpatient clinic. These findings were not reproduced in the other studies [6, 9], probably due to insufficient sample size. The strategy of targeting for intervention only those patients who are at higher risk can improve cost-efficacy. In fact, in the described context, the time spent by health professional in training patients in the intervention group was smaller than that used for the treatment of preventable ulcers in the control group. The approach used, based on interactivity and practical demonstrations, and aimed at improving skills, rather than formal knowledge, provided interesting clinical results.

Some limitations of the present study should be recognized. This is a single-center trial, performed by highly trained health professionals working in a diabetic foot clinic; the reproducibility of this program in a different setting should be verified. The cost of drugs and materials used for the treatment of ulcers was not assessed, leading to an underestimation of the direct costs in the control group. Although patients' knowledge was improved by the intervention, no data were collected on patients' skills or actual behaviors during follow-up. In addition, the therapeutic effects of patient education tend to fade with time [12]; the durability of the beneficial effects of this program needs to be formally tested in a study with a longer follow-up.

Despite these limitations, the proposed intervention appears to provide a sustainable and effective approach to targeted education for diabetic foot ulcer prevention.

## Figures and Tables

**Table 1:** Baseline demographic and clinical characteristics of the patients enrolled.

	Standard care	Educational program	*p*
Number	60	60	—
Gender (women, %)	28 (46.7)	20 (33.3)	0.14
Age (years)	69.4 ± 11.3	72.0 ± 8.9	0.11
Duration of diabetes (years)	15.9 ± 11.2	14.2 ± 12.4	0.48
BMI (Kg/m^2^)	30.0 ± 5.6	29.4 ± 4.7	0.61
Waist circumference (cm)	106.4 ± 13.9	104.2 ± 11.1	0.51
HbA1c (%)	7.3 ± 1.4	7.4 ± 1.3	0.86
Systolic pressure (mmHg)	139 ± 19	136 ± 17	0.41
Diastolic pressure (mmHg)	75 ± 10	79 ± 16	0.24
Smokers/ex-smokers (%)	20 (33.3)	21 (35)	0.59
Charlson's comorbidity score	2.9 ± 2.8	3.0 ± 2.7	0.87
*PIN questionnaire score*	20 [16; 22]	19 [16; 20]	0.65
Medical history (%)			
Peripheral artery disease	10 (16.7)	5 (8.3)	0.17
Neuropathy	48 (80.0)	50 (83.3)	0.89
Previous ulcers	6 (9.9)	7 (11.7)	0.49
Foot abnormalities^*∗*^	6 (9.9)	3 (4.9)	0.37
Retinopathy	4 (6.7)	10 (16.7)	0.088
Chronic renal failure^‡^	6 (10.0)	7 (11.7)	0.77
Cardiac disease^†^	18 (30.0)	18 (30.0)	>0.99
Chronic heart failure	6 (10.0)	5 (8.3)	0.75
Cerebrovascular disease^††^	6 (10.0)	4 (6.7)	0.51
Nonmetastatic malignancies	4 (6.7)	5 (8.3)	0.73
Treatment (%)			
Insulin	12 (20.0)	17 (28.3)	0.29
Antihypertensive	46 (76.7)	50 (83.3)	0.71
Statin	28 (46.7)	25 (41.7)	0.67
Antiaggregant/coagulant	50 (83.3)	52 (86.7)	0.78

^*∗*^In absence of neuropathy; data are expressed as number (%) and mean ± SD; ^†^previous myocardial infarction and/or angina pectoris; ^††^previous stroke or transient ischemic attack; ^‡^creatinine >1.2 mg/dL.

**Table 2:** Selected clinical parameters at 6-month follow-up.

	Standard care	Educational program	*p*
Number	60	60	—
BMI (Kg/m^2^)	30.1 ± 5.7	29.5 ± 4.6	0.29
HbA1c (%)	7.3 ± 1.4	7.1 ± 1.2	0.37
Systolic pressure (mmHg)	136 ± 15	137 ± 18	0.62
Diastolic pressure (mmHg)	77 ± 11	78 ± 16	0.65

Data are expressed as number (%) and mean ± SD.

## Appendix

### Description of the Educational Program

The educational program was composed of two sections.

*(1) Frontal Lessons*. This part was aimed at providing general information on the risk factors for foot ulcerations, in particular, peripheral neuropathy and artery disease. Symptoms and signs of these two latter complications were fully described from medical doctors and trained nurses, pointing out the relevant role of glycemic control, lipid profile, blood pressure, smoking habits, and so forth, on the risk of foot lesions. Some other important local factors, such as mechanical abnormalities (joint mobility, high plantar pressure, callus, etc.) and fungal infections, were also discussed, and the usefulness of daily foot inspection, foot hygiene, adequate orthopedic shoes, and orthotic footwear/inserts, and so forth was outlined.

Patients were instructed on how to check regularly their feet (using a mirror or asking for someone else's help), looking for possible lesions. The following information was also provided:

- Ulcers: minor scrapes or cuts that heal slowly, or sores from unfit shoes, can become infected, causing ulcers.
- Dry skin: patients were advised to use moisturizing soaps and hydrating lotions/oils, without using them between toes.
- Blisters: they can be a sign of unfit shoes, and they should not be opened (for the risk of infection).
- Corns/calluses: they should be gently removed with an emery board or pumice stone, without using any kind of blade.
- Ingrown toenails: toenails must be trimmed regularly.
- Discolored/yellowed toenails: they are a possible sign of a fungal nail infection.
- Redness, warmth, swelling, or pain: they are possible symptoms of inflammation and infection; medical advice should be immediately sought in this case.
- Blue or black skin color: they are possible symptoms of critical ischemia, needing immediate medical contact.

At the end of this formal lesson, patients were allowed to participate in an interactive discussion.

*(2) Interactive/Practical Section*. In this section, healthcare professionals showed some practical actions to reduce the risk of foot ulcers:

- Footbath: one patient was asked to prepare an adequate (in his/her opinion) foot bath and to wash his/her feet. Based on the performance of the patient, the whole group was asked to discuss the procedure. The aim of this exercise was the acquisition of the following rules:
  - Use of warm water.
  - Choice of mild soaps.
  - Accurate drying of feet, including interdigital areas.
- Using a pen, patients were asked to trace the outline of their entire foot on a sheet of paper and to put it inside the shoe they wore. The aim of this exercise was that of focusing the patients' attention on the need for fit and comfortable shoes. The choice of shoes was then discussed with the whole group.
